# An Unobstructive Sensing Method for Indoor Air Quality Optimization and Metabolic Assessment within Vehicles

**DOI:** 10.3390/s20247202

**Published:** 2020-12-16

**Authors:** Yue Deng, Mark Sprowls, S. Jimena Mora, Doina Kulick, Nongjian Tao, Hugo Destaillats, Erica Forzani

**Affiliations:** 1 School of Engineering for Matter, Transport, and Energy, Arizona State University, Tempe, AZ 85281, USA; catherinedeng1015@gmail.com (Y.D.); mark.sprowls@asu.edu (M.S.); smora2@asu.edu (S.J.M.); 2 Center for Bioelectronics and Biosensors, Biodesign Institute, Arizona State University, Tempe, AZ 85281, USA; 3 Mayo Clinic, Scottsdale, AZ 85054, USA; Kulick.Doina@mayo.edu; 4 School of Electrical, Energy and Computer Engineering, Arizona State University, Tempe, AZ 85281, USA; njtao@asu.edu; 5 Lawrence Berkeley National Laboratory, Indoor Environment Group, Berkeley, CA 94720, USA; hdestaillats@lbl.gov

**Keywords:** indoor air quality, carbon dioxide accumulation, metabolic rate, energy expenditure, passive sensing

## Abstract

This work investigates the use of an intelligent and unobstructive sensing technique for maintaining vehicle cabin’s indoor air quality while simultaneously assessing the driver metabolic rate. CO_2_ accumulation patterns are of great interest because CO_2_ can have negative cognitive effects at higher concentrations and also since CO_2_ accumulation rate can potentially be used to determine a person’s metabolic rate. The management of the vehicle’s ventilation system was controlled by periodically alternating the air recirculation mode within the cabin, which was actuated based on the CO_2_ levels inside the vehicle’s cabin. The CO_2_ accumulation periods were used to assess the driver’s metabolic rate, using a model that considered the vehicle’s air exchange rate. In the process of the method optimization, it was found that the vehicle’s air exchange rate (λ [h^−1^]) depends on the vehicle speeds, following the relationship: λ = 0.060 × (speed) − 0.88 when driving faster than 17 MPH. An accuracy level of 95% was found between the new method to assess the driver’s metabolic rate (1620 ± 140 kcal/day) and the reference method of indirect calorimetry (1550 ± 150 kcal/day) for a total of N = 16 metabolic assessments at various vehicle speeds. The new sensing method represents a novel approach for unobstructive assessment of driver metabolic rate while maintaining indoor air quality within the vehicle cabin.

## 1. Introduction

Vehicle collisions have historically been one of the leading causes of preventable death in the United States. Increased legal focus on punishing distracted and/or inebriated drivers has had a surprisingly small effect on the annual number of vehicle-related casualties, as evidenced by the 7-year high in fatalities caused by vehicle collisions within the United States in 2016 [1]. Vehicle collisions and resulting injury are a serious burden to the U.S. healthcare system, with an estimated annual cost of $99 Billion [2]. Recent developments in the field of air quality control have shown that CO_2_ buildup to 1000 ppm and above frequently occurs in mid-size vehicles, especially with the use of recirculation mode [3,4,5,6,7]. CO_2_ is produced as a natural product of human metabolism and its accumulation within closed environments with insufficient ventilation can lead to accumulation. CO_2_ overexposure has been associated with reduced cognitive performance. These effects have been shown to be present at or above 1000 ppm CO_2_, and worsen significantly when CO_2_ concentration reached 2500 ppm or higher in laboratory settings [8,9,10,11]. A recent study has evaluated the effect of similar CO_2_ levels on commercial airplane pilots performing maneuvers in a flight simulator showing a correlation of elevated CO_2_ concentrations with lower flight maneuver success, independent of ventilation [12]. As such, CO_2_ concentration monitoring within vehicles may be of great value to public health from a safety perspective.

On a separate area, the air exchange rate (λ, referred to as AER or ACH in other works) from a vehicle’s cabin have been extensively studied, as well as the effect of driving speed on air exchange rate [13,14,15,16,17]. A major factor leading to CO_2_ accumulation in vehicle cabins is the use of recirculation (RC) mode, since most of the CO_2_ exhaled by occupants is not vented outside when this ventilation setting is selected. For comfort and energy efficiency purposes, in many vehicles the RC mode is automatically turned on when the air conditioning is operating. The presence of additional occupants within a vehicle further accelerates CO_2_ buildup [18]. CO_2_ accumulation patterns have also been simulated using training data from a large set of vehicles in [4], which provides a guide for predicting maximum CO_2_ concentrations during drives of various lengths while using RC mode. However, other works rely on air exchange rate measurement in vehicles using tracer gas techniques, previously SF_6_ has been documented in vehicles for this purpose [14], or on steady-state measurements of CO_2_ concentration [13], which can be inconvenient. We report here a method of λ evaluation from unsteady-state (transient) CO_2_ accumulation data that poses significant advantages over both methodologies mentioned above. The new method does not require the use of exogenous trace gas and allows accumulation of CO_2_ at the steady-state level, which can reach dangerous levels (e.g., 2548 ppm for a single subject driving at 32 km/h [13]).

The present work strives to investigate the use of an unobstructive sensing method relying on unsteady-state (transient) CO_2_ accumulation measurement for maintaining vehicle cabin’s indoor air quality and assessing the driver’s metabolic rate, using a previously derived model [19] and pre-calibrated car’s air exchange rate as a function of driving speed. This system could potentially leverage the wealth of data collected and resulting general linear model in [13], which takes into account a wide fleet of vehicles of various ages and manufacturers to estimate λ for a given vehicle based on manufacturer and age.

In the field of nutritional medicine, CO_2_ production has a close mathematical relationship with human metabolic rate, referred to as energy expenditure (EE), and expressed in kcal/day [20]. EE is the key physiological metric used to take a medical intervention for clinically obese patients since it allows medical doctors to make recommendations for calorie consumption on a given day based on their patient’s rate of energy consumption [21]. This study probes the feasibility of EE determination by quantifying the volumetric production rate of CO_2_ (VCO_2_), expressed in mL/min using only ambient and non-wearable sensors. Measuring EE based on ambient CO_2_ production using only one portable CO_2_ sensor within a vehicle is a promising approach. Methods for ambient VCO_2_ measurement do exist, but depend on CO_2_ measurements in inlet and outlet ducts in a room with controlled mechanical ventilation, making the system’s installation too cumbersome for widespread usage [22]. Other medical devices require the use of breathing hardware that prevents free living EE determinations, are time consuming and discourage repeated measurements [23]. This study describes initial step towards validation of a medical device for EE quantification in a controlled indoor microenvironment. Overall, the development of this device fits into a larger growing trend in the field of cutting edge biomedical diagnostics which in coming years may be largely characterized by what can be described best as “minimally invasive IoT (internet of things) medical devices and biosensors” [24,25,26,27]. The basic premise of this growing field of biomedical diagnostics is to engineer minimally invasive medical devices that allow for clinically relevant biometrics to be collected repeatedly on the order of days, weeks, or months in a manner that is minimally obtrusive (i.e., no blood sampling, minimal user time commitment for collection, ambient sensing, use of wearables) and provides relevant health biometrics to clinicians without sacrificing accuracy (or partially mitigating lower accuracy with massive sample size that invasive devices fail to feasibly achieve for the everyday patient). However, IoT sensors are not only promising for the field of medical devices, but also for the purpose of environmental data collection and also, in some cases, (such as the method this work proposes) artificially intelligent closed-loop actuation control. Following this line of reasoning, the ideal intelligent vehicle air quality optimization system could be potentially multiplexed with a sensing approach that also considers outdoor toxic pollutants such as NO_2,_ and, therefore, an array of NO_2_ and CO_2_ using IoT sensors could be placed together to make intelligent decisions in the utilization of RC mode or regular air conditioning (AC) mode depending on air quality conditions and current concentrations of both chemical species to minimize driver risk. With recent developments in triboelectricity-based NO_2_ sensors [28,29], this is a very real possibility and may be the focus of future works.

A comprehensive assessment of CO_2_ levels inside a car cabin was conducted, including experimental characterization and model simulation. Our team studied the CO_2_ production rate by a single occupant (driver) and the cabin air exchange rate (λ) under one fan condition and driving speeds. These results allowed for the assessment of the driver’s energy expenditure. A box model was used to predict the CO_2_ concentration profile within a vehicle under various conditions.

## 2. Materials and Methods

### 2.1. Experimental Characterization

#### 2.1.1. Experimental Setup

A set of tests was carried out by a single investigator driving a vehicle on residential streets and highway within the greater Phoenix area, Arizona, USA, between August 2017 and July 2018. The vehicle tested was a 2012 Hyundai Elantra. Its age at the time of the tests (5–6 years) and interior volume (110 ft^3^, or 3.1 m^3^) (“2012 Hyundai Elantra Features & Specs,”) are very close to the median values for the current US gasoline-fueled light duty vehicle fleet (8 years and 110 ft^3^, respectively) [4]. The study was approved by Arizona State University Institutional Review Board (IRB protocol # STUDY00006547). The subject (car driver) was a healthy 27-year-old female. The test subject provided written informed consent before participating in the study.

#### 2.1.2. Sensing System

The custom-made measurement system consisted of a Telaire^®^ 7001 CO_2_ sensor (Onset Corp, Bourne, MA, USA) and a HOBO^®^ temperature and humidity sensor (Onset Corp, Bourne, MA, USA), as described in our previous publication [19]. The system was calibrated with CO_2_ gas samples of different concentrations in the range of 0–3000 ppm. In addition, prior to each experiment, a quality control procedure was applied for every measurement assuring the reading of outer CO_2_ concentration roughly matched the expected value (~400 ppm), to ensure the outdoor environment was free of any urban or biogenic source.

#### 2.1.3. Measurements and Sensing Methods

During the test, the sensing system was placed on top of the front passenger seat, approximately one meter from the driver. Real-time CO_2_ concentration, temperature, and humidity were recorded with a resolution of 1 s^−1^. All tests were conducted at times of low traffic for consistent driving speed and to avoid introducing CO_2_ by air exchange with the outside environment, since CO_2_ effluent from surrounding vehicle exhaust could enter the vehicle cabin as car exhaust is a form of highly concentrated CO_2_ (relative to regular atmospheric levels). Additionally, it is reasonable to postulate exhaust streams are at a higher pressure than the internal car cabin pressure (due to engine heat, among other factors) and are high in CO_2_ concentration since they are fuel effluent which would serve as a source of error for observed CO_2_ accumulation patterns if it were not controlled by driving at low traffic times.

**Figure 1:**
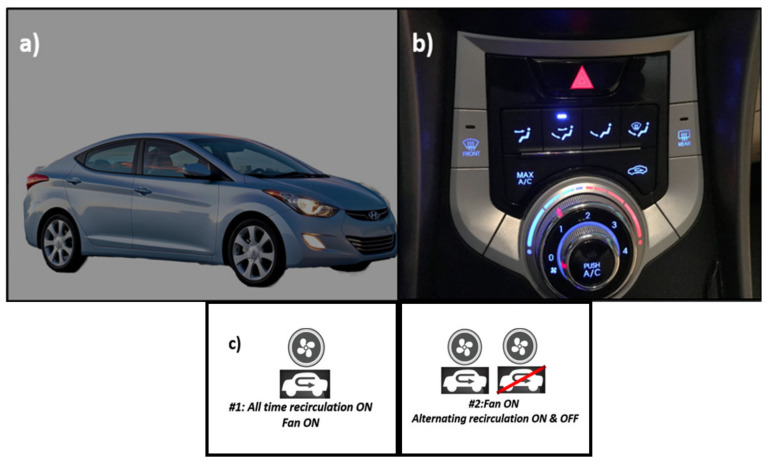
(**a**) Vehicle used in this study; (**b**) AC control panel; (**c**) Two different testing conditions, see text for detail.

A picture of the vehicle model used in the study is shown in Figure 1a. Figure 1b shows the AC dashboard, including fan speed level and an independent control to select between RC or air exchange mode.

Two different ventilation methods were used with the car windows closed, as described in Figure 1c:

Method #1–Continuous RC (recirculation) mode: In this method, the RC mode was on and the fan was kept at level 1 during the tests. Five different driving speeds were tested under this condition: 0 MPH (miles per hour), 15–17 MPH, 33–35 MPH, 48–50 MPH and 68–70 MPH. To achieve different level of speed, the tests were conducted on residential roads (Broadway and Rural road in Tempe, AZ, USA) and highway (AZ Loop 101, Loop 202 and Interstate 10 in Arizona) accordingly. The speed log of each test was recorded with the RunKeeper^®^ app (ASICS Digital, Boston, MA, USA). The test was stopped once the cabin CO_2_ concentration reached 2000 ppm or if the test had lasted 0.8 h. These tests were performed 3 times at each speed. One growth curve is provided at each speed as an example.

Method #2–Continuous ventilation and intermittent RC mode: In this method, the RC Mode is intermittently ON and OFF with the continuous ventilation ON at a fan level 1. The RC was turned OFF and actuated based on the CO_2_ levels inside the vehicle’s cabin. These levels were between baseline level of ~450 ppm for fresh air and threshold levels of 1000–1100 ppm for air resulting from the CO_2_ accumulation due to the driver’s breathing. In other words, the RC mode was initially on and was turned off for five minutes once the cabin CO_2_ concentration reached around 1000–1100 ppm, to allow for air exchange with the external environment and reduction of CO_2_ levels within the vehicle cabin. Under this condition, four different driving speeds were tested: 0 MPH, 15–17 MPH, 33–35 MPH and 65–68 MPH. The roads taken for this set of tests were the same as condition #1. Each test was performed within a single 1 h span. The RC mode was switched ON/OFF 4 times total within the hour for each speed tested. This rendered 4 separate growth curves (and, therefore, 4 separate metabolic rate measurements). 

#### 2.1.4. Local Concentration Gradients within Vehicle

A previous computational fluid dynamics (CFD) study [30] has shown that, in theory, CO_2_ concentration gradients [ppm] within the occupied vehicle do not vary by greater than an order of magnitude, in most cases and except at geometric boundary conditions. In our experimental conditions, the effects of mixing and the presence of concentration gradients were examined with the vehicle at rest, showing little variability.

### 2.2. Simulation and Data Analysis

#### 2.2.1. Carbon Dioxide Accumulation Analysis

Ji et al. [31] has previously considered a model for human-generated CO_2_ accumulation. However, the model applies only to conditions of a human inside a perfectly sealed small chamber, with no leakage between the chamber and its surroundings. Using a model described in our previous study and which accounts for air leakage in the environment, which are representative of realistic human free-living conditions [19], a computational simulation was used to predict CO_2_ accumulation patterns for various scenarios based on the solution of a total differential equation for changes in CO_2_ concentration with respect to time. The developed model was simulated using MATLAB^®^ (MathWorks Inc., Natick, MA, USA) and assumes that $\frac{d[CO_{2}]}{dt}$ is first order with respect to CO_2_ concentration and 0th order with respect to CO_2_ generation (i.e., the amount of CO_2_ generated by occupants does not depend on CO_2_ concentration). The solved differential equation for CO_2_ generation is as follows:

(1) $$[CO_{2}]={ [CO_{2}]}_{0}+\frac{K_{gen}}{\lambda }\left(1-e^{-\lambda t}\right)\;+\;{ [CO_{2}]}_{i}e^{-\lambda t}$$

where ${ [CO_{2}]}_{0}$ is the ambient CO_2_ concentration (~400 ppm), ${ [CO_{2}]}_{i}$ is the difference between the initial CO_2_ concentration at the beginning of the fitted curve and the baseline CO_2_ concentration, $k_{gen}$ is CO_2_ generation rate with units of ppm CO_2_ h^−1^, and λ is the air exchange rate in h^−1^. The volumetric production rate of CO_2_ (VCO_2_ [mL/min] by the vehicle’s occupant can be calculated as follows and is simply derived from the ideal gas law and a mass balance on CO_2_ within the cabin:

(2) $$VCO_{2}=k_{gen}\;\times \;V_{Room}\;\times \;CF_{STPD}/60$$

where VCO_2_ is the subject’s volumetric production of CO_2_ [mL/min], V_Room_ is the volume of the vehicle cabin [mL] (taken from manufacturer-listed vehicle specifications), and CF_STPD_ [dimensionless] is a correction factor to correct the VCO_2_ at ambient temperature and pressure conditions (ATP) to standard temperature, pressure, and dry conditions (STPD). The correction factor was calculated as follows:

(3) $$CF_{STPD}\;=\;\frac{P_{bar}\;-\;P_{H20}}{760}\;\times \;\frac{273}{T+273}$$

#### 2.2.2. Effect of Car Occupant’s Metabolic Rate

The proportion of a person’s CO_2_ production rate (VCO_2_) to O_2_ consumption rate (VO_2_) is known as the respiratory quotient (RQ) and its value depends on the ratios of metabolized energy sources within a person’s body (e.g., RQ for carbohydrate metabolism = 1.0; fat metabolism = 0.71; protein metabolism = 0.82) [32]:

(4) $$RQ\;\left(Respiratory\;Quotient\right)\;=\;\frac{VCO_{2}}{VO_{2}}$$

The relationship between RQ, VCO_2_, and energy expenditure (EE) has been studied extensively and is the basis of the field of indirect calorimetry. The fundamental equation that links these 3 parameters is known as Weir Equation and it is shown below [33]:

(5) $$EE\;\left(\frac{kcal}{day}\right)\;=\;1.44\;\times \; [3.941\;\times \;\frac{VCO_{2}}{RQ}\;+\;1.11\;\times \;VCO_{2}]$$

For individuals following diets with relatively equal amounts of carbohydrates, protein, and fats, an RQ equal to or near 0.85 should be expected [34,35,36]. In this case, Equation (5) can be simplified to the following:

(6) $$EE\;\left(\frac{kcal}{day}\right)=\;8.273\;\times \;VCO_{2}$$

#### 2.2.3. Simulation Parameters

It is important to mention that EE assessment is solely possible when we have a single occupant in the vehicle. For multiple occupants, VCO_2_ production will be representative of the driver + occupants. The multiple occupants could be detected with pressure sensors in the seat, and a combination of pressure sensors could discriminate if the occupant is a subject or an object. Under these conditions, an averaged EE value per occupant could be assessed. However, the indoor air quality aspect of the presented approach would be more relevant to assure the safety of the indoor air inside the cabin. Therefore, for the purpose of informing public policy that governs domestic roadways, it has great utility in the sense that this information can be used to build simulations detailing the CO_2_ concentration in various accumulation conditions (as done in Section 2.2.3 below) that are useful in assessing driving safety relative to CO_2_ concentration within the vehicle cabin.

The simulation presented in this work has been developed to extend the experimental findings introduced in this work’s assessment to several other conditions that could not be tested. The model estimated the car cabin volume to be 3.1 m^3^, a baseline CO_2_ concentration of 400 ppm, a λ of 1 h^−1^ (unless indicated otherwise), a linear relationship between λ and car speed (validated by experimental findings presented in this work), a linear relationship between CO_2_ generation and the number of occupants within the vehicles, and an occupant energy expenditure *EE* = 1700 kcal/day (unless indicated otherwise).

## 3. Results

### 3.1. Experiments under Method #1—Continuous RC (Recirculation) Mode

**Figure 2:**
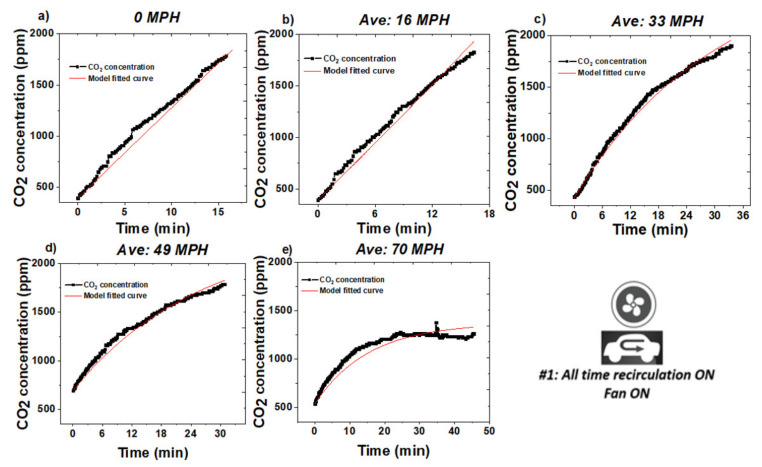
CO_2_ concentration inside the vehicle cabin under different driving speed, all-time recirculation (RC) on mode. (**a**) 0 MPH (parked); (**b**) 16 MPH; (**c**) 33 MPH; (**d**) 49 MPH; (**e**) 70 MPH.

The purpose of this set of experiments was to examine the intrinsic air exchange condition according to our group’s derived model and explore its relationship with driving speed. Figure 2 summarized the results together with the average recorded speed, while real-time speed data are shown in Figure S2 (Supplementary Materials). Three experiments were performed at each speed on different days as replicate measurements.

We hypothesize that the increased leakage at higher driving speeds is due to the pressure difference between inside the cabin and the outside environment, generated by the vehicle movement, and Bernoulli principle of differential negative pressure causing a net flux of air out to the vehicle and into the surrounding environment due to the vehicle’s velocity. Thus, it is reasonable to assume that at 0 MPH (parked condition), there is significantly reduced leakage due to a negligible pressure difference. As summarized in our previous publication [19], under this condition, the CO_2_ concentration profile should follow a linear relationship. Applying linear fitting to 0 MPH data, we determined the actual CO_2_ generation rate to be *K_gen_* = 5344 ± 66 ppm/h. Additionally, to show that the vehicle’s CO_2_ concentration is independent of engine operation (which of course, can be a source of CO_2_), an experiment was performed to assess the cabin’s CO_2_ concentration with the vehicle’s engine on, the vehicle stationary, unoccupied, and with recirculation mode on. Under these conditions, it could be clearly observed that there is no CO_2_ accumulated within the vehicle’s cabin from engine exhaust. This error mitigation assessment is shown in Figure S1 of the Supplementary Materials. 

In each of the replicate experiments, five tests at different speeds were carried out consecutively in a 3 h period, during which the driver remained seated at the wheel. For that reason, the driver’s energy expenditure was considered to be relatively constant over the course of the experiment. Using the experimentally determined *K_gen_* value and Equation (1), we determined the effective air exchange λ for each speed.

**Figure 3:**
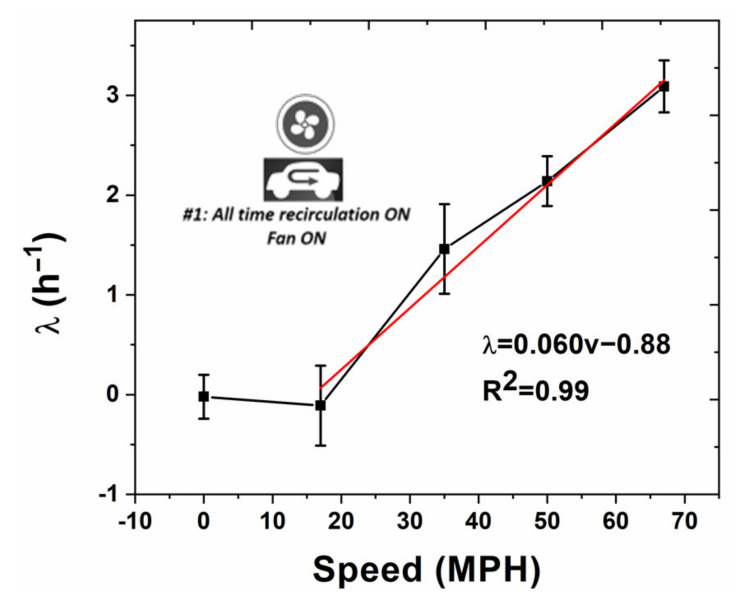
Relationship between the effective air exchange rate (λ) and driving speed (ν). Red line indicates linear regression curve above 17 MPH.

The model fitted λ results are shown in Figure 3. The error bars for each speed is the standard deviation from three replicates. It is observed that in the range of 0–18 MPH, λ is close to 0. Thus, we define that when the speed is less than 17 MPH, the intrinsic air exchange is negligible. By applying linear fitting for speeds > 17 MPH, we could calculate the relationship between λ and driving speed, as presented in Figure 3. The regression coefficient (R^2^) equals 0.99, indicating a strong correlation between the two parameters. As can be observed in the Supplementary Materials for this work (Figure S2), vehicle speed did not remain constant during each nominal driving speed. In fact, there was a moderate degree of variance. However, the average of the driving speed allowed the output variables of λ or EE to be assessed, depending on the driving condition. Therefore, from the collected data, it is not unreasonable to hypothesize that “the collected data suggests that even when there is a moderate degree of variation within vehicle velocity, the proposed model still remains.

This result is also consistent with other reports showing that higher driving speeds resulted in slower CO_2_ concentration growth profiles [4]. However, quantitatively, results differed from those predicted in the model described in [13], where the predicted λ’s were at least 2x greater than the results observed in the present study. Potential reasons for this discrepancy are the car manufacturer (Korean manufacturer adjustment not available for the model, as such, Japanese adjustment was used instead) and also that no vehicles from a 2012 fleet were tested in Fruin’s 2011 work. Additionally, in Fruin’s 2011 work, the technique for λ (referred to as AER in Fruin’s work) assessment relied on analysis of concentration equilibrium conditions, whereas, the present work relies on analysis of unsteady-state CO_2_ growth curves. In another work [14], characterizing λ using the constant injection SF_6_ tracer gas technique, observed air exchange rates were within the normal range for the vehicle tested in the present study, indicating that the CO_2_ analysis technique used in the present work coincides well with previous literature values for λ assessment using a different technique. One significant limitation of the present work is that wind speed and outdoor concentration were not tested as in [13], as these factors can have a significant effect, also shown in [14,15].

### 3.2. Experiments under Method #2—Continuous Ventilation and Intermittent RC Mode

Under this condition, the CO_2_ concentration was kept under 1100 ppm by alternating RC on and off, and the fan ventilation level at 1. Typically, in one measuring cycle, RC mode would be left on for about 5 min and then turned off for 5 min once the CO_2_ concentration reached 1100 ppm. The aim was to assess energy expenditure during the growth periods of CO_2_ buildup, and validate the model (by comparing using *EE* measurements taken from a reference instrument) at a condition where recirculating cabin air (RC mode) and outside air ventilation were alternated. Since regular, non-RC mode ventilation consumes more of the vehicles fuel in operation in comparison with RC mode [37], this condition is a practical solution to assure good quality levels in the air inside the car cabins while minimizing the fuel efficiency losses due to car cabin ventilation. In addition, these experimental conditions allow for the assessment of energy expenditure with the added benefit of being able to perform multiple *EE* measurements within a single trip, which improves the system’s accuracy with respect to assessment of the driver’s average energy expenditure. This is the true highlight of the observed data, as it is already known that turning off RC mode will mitigate CO_2_ buildup within vehicles.

**Figure 4:**
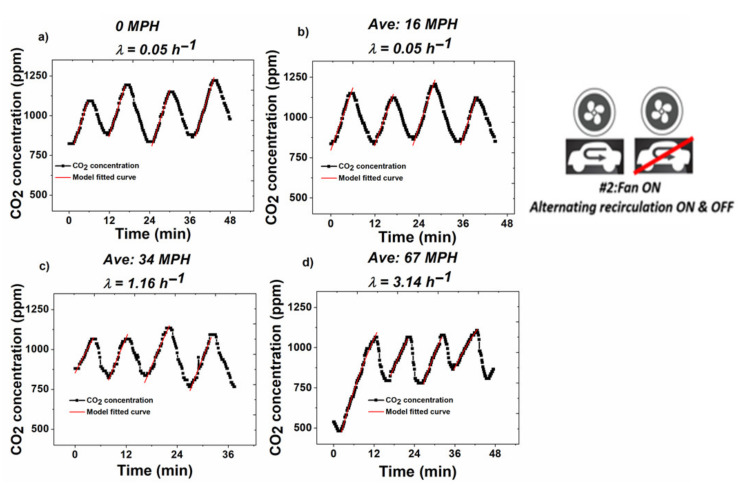
Real-time CO_2_ concentration profile and the model fitted curve (red) under different driving speed. (**a**) 0 MPH; (**b**) 16 MPH; (**c**) 37 MPH; (**d**) 64 MPH.

Real-time CO_2_ profiles are shown in Figure 4. The model was applied for a λ of 0.05 h^−1^, which is a non-null but negligible value for speeds lower than 18 MPH. The assessment of this condition was based on the experimental observation that at parked and at low speeds, the air exchange rate between the vehicle cabin and the environment was negligible. For speeds greater than 18 MPH, the λ was calculated from the regression equation shown in Figure 3 and used in Equation (1) to determine the real *K_gen_* for each CO_2_ growth period test cycle obtained during the periods of 36–48 min driving at a certain speed. Real-time speed data are shown in Figure S3 (Supplementary Materials).

**Figure 5:**
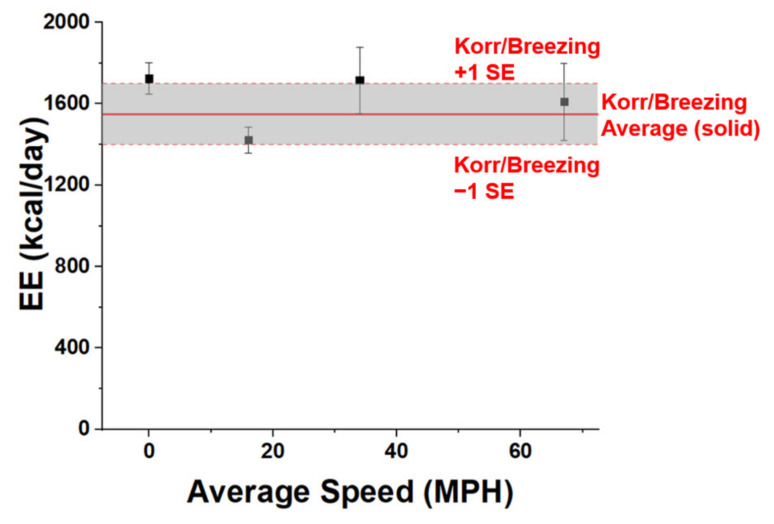
Energy Expenditure (*EE*) estimates generated from experimental data shown in Figure 4 (shown as black points with error bars) and corresponding reference instrument (Korr Reevue^TM^ and Breezing Pro^TM^) measurements shown as horizontal redline (average) ±1 standard error.

VCO_2_ values calculated using Equation (1) for fitting CO_2_ accumulation data and then decomposing the K_gen_ term to provide an estimate for VCO_2_ via equation 2, were then used to calculate *EE* using Equation (4) for each growth cycle, using results from four cycles obtained at each speed, and averaged for that speed. For these four tests, the *EE* value ranged from 1420 kcal/day to 1730 kcal/day with an average of 1620 ± 140 (kcal/day). The results are shown in Figure 5 for the averages with their corresponding standard error. The coefficient of variation from four tests remained at 8.6%, indicating there was no significant *EE* change during the test at each driving speed (36–48 min). This is consistent with our hypothesis in Section 2.1 that the participant remained in a stable metabolic state over the duration of the driving tests. Another important finding was that energy expenditure fluctuated randomly within a relatively narrow range of values. The random variability (±15%) reflects the typical clinical variability expected for an energy expenditure measured at free-living conditions [38]. These results confirm that energy expenditure is independent from vehicle velocity.

Energy expenditure measured during driving tests were compared with those determined with conventional instrumentation. The subject’s energy expenditure (or metabolic rate) while sitting in a computer and working was measured by indirect calorimetry using two different instruments: the desktop Korr Reevue^TM^ (www.korr.com, Salt Lake City, UT, USA) and the Breezing Pro^TM^ (https://breezing.com/, Tempe, AZ, USA), obtaining an average of (1550 ± 150) kcal/day for 10 measurements utilizing both indirect calorimetry instruments (5 readings each). This represents only ~4% difference in comparison with our calculated *EE* value. It should be noted that the participant did not perform any intense activity on the days of tests, since strenuous exercise increase a person’s instantaneous energy expenditure [38]. The results demonstrated that this model could be used to determine *EE* of drivers, as the difference between mean values determined with each method was lower than the relative error for each of them. However, it is of course important to note that the subject did not perform the reference instrument *EE* assessments simultaneously while driving, due to safety concerns regarding vehicle operation.

### 3.3. CO_2_ Concentration Profile Modeling

**Figure 6:**
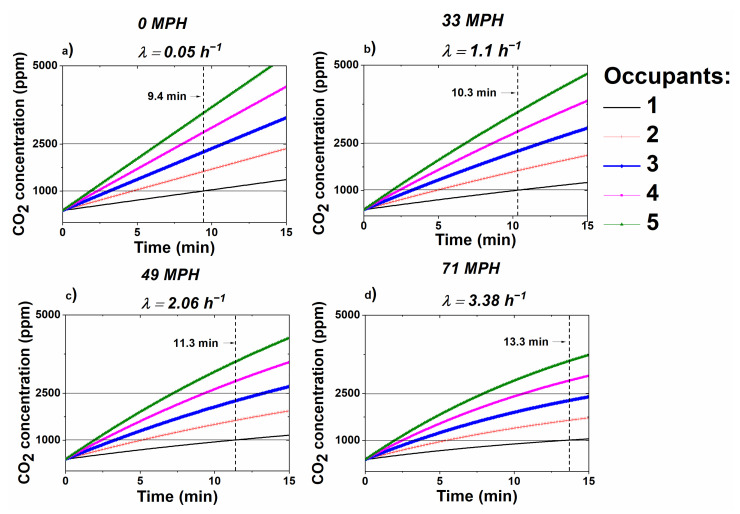
Modeled CO_2_ concentration profile for various driving speeds and number of occupants within vehicle. (**a**) 0 MPH; (**b**) 33 MPH; (**c**) 49 MPH; (**d**) 71 MPH.

To expand upon the experimental results shown in the previous sections of this work, a computational simulation was developed using MATLAB^®^ to generate model CO_2_ growth profiles under various conditions that were not investigated experimentally in this study. Figure 6 simulates CO_2_ concentration growth profiles inside a car cabin (RC mode on) with different number of occupants under various speeds. A horizontal line has been drawn at both 1000 and 2500 ppm with a label to indicate the corresponding time at which a vehicle with a single occupant reaches the aforementioned CO_2_ concentration, used as a reference from recent cognitive performance studies [8,9,10,11]. The simulation predicts that CO_2_ levels within the car cabin reach 1000 ppm for a single occupant in less than 15 min with RC mode on. CO_2_ accumulation is significantly higher for car cabins where there is more than one occupant; this is clearly evidenced in the simulation’s output where CO_2_ levels exceeding 2500 ppm are reached in under 15 min when the vehicle is occupied with at least three occupants, regardless of vehicle speed.

**Figure 7:**
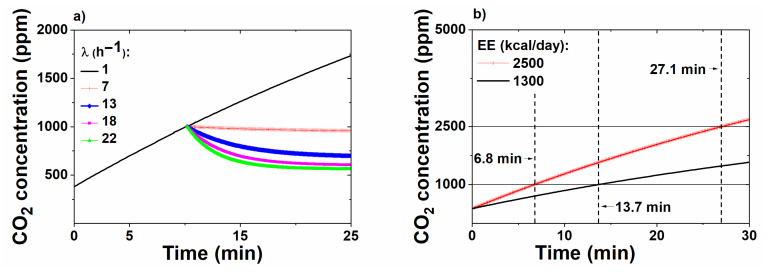
(**a**) Model CO_2_ concentration profile showing effect of air exchange rate on CO_2_ level; (**b**) Modeled CO_2_ concentration profile showing effect of metabolic rate on growth rate.

Figure 7a shows how a CO_2_ profile can be affected by changing the effective air exchange rate from 1 to a higher value, up to 22 h^−1^, e.g., by alternating the RC mode on and off, and/or opening windows. It is worth noticing that although the lower λ values of 1–3 h^−1^ represent the values assessed in the experimental results of this work, which involved the car windows being closed, higher λ could be obtained with car windows opened and were included since this work represents a generic model that can be applicable to multiple other driving conditions.

Figure 7b demonstrates the effect of occupant metabolic rate on CO_2_ accumulation within a vehicle. This parameter has a substantial influence on the CO_2_ concentration growth profile. A driver with a relatively high *EE* of 2500 kcal/day will reach a CO_2_ concentration of 1000 ppm in just 6.8 min, as compared with virtually twice as long (13.7 min) for a driver spending only 1300 kcal/day. The high-*EE* driver can reach a CO_2_ concentration of 2500 ppm in less than half an hour.

## 4. Conclusions

In the present work, we characterized transient CO_2_ buildup and the vehicle air exchange rates under different driving speeds. Most importantly, we developed a new method to assess the air exchange rate inside the car cabin under non-steady conditions by using the driver’s energy expenditure rate as a known variable. This new method enabled the assessment of the air exchange rates and the car speed function, which further proved to be useful to assess the driver’s energy expenditure repeated times under specific conditions of an on/off alternating ventilation. The results showed a good regression fitting (R^2^ > 0.99) on a real-time CO_2_ concentration profile and accurate calculation for a single driver’s *EE* (4.4% difference with respect to determination using standard methods). Simulation results based on experimental data were also presented. The simulation serves to predict CO_2_ accumulation patterns due to various factors such as number of occupants and occupant energy expenditure. Overall, the investigations of this work allowed for the creation of an intelligent and unobstructive sensing method for maintaining vehicle cabin’s indoor air quality and assessing the driver’s metabolic rate.

## Supplementary Materials

The following are available online at https://www.mdpi.com/1424-8220/20/24/7202/s1, Figure S1: Temperature, relative humidity (RH), and CO_2_ concentrations versus time within a car while driving with the ventilation system closed, and the driver breathing in and out of the car cabin via a tubing system that preclude from exhale carbon dioxide buildup; Figure S2: Raw CO_2_ monitor results together with real-time speed log for condition #1; Figure S3: Raw CO_2_ monitor results together with real-time speed log for condition #2.
