# Supplementary material for: An Unobstructive Sensing Method for Indoor Air Quality Optimization and Metabolic Assessment within Vehicles
Source: Sensors (Basel). 2020 Dec 16;20(24):7202. doi: 10.3390/s20247202 (PMC7766572; doi:10.3390/s20247202)
Supplement: Supplementary file 1 [file sensors-20-07202-s001.pdf]

sensors

Article

An Unobstructive Sensing Method for Indoor Air Quality Optimization and Metabolic Assessment within Vehicles

Yue Deng [1,2,†], Mark Sprowls [1,2,†], S. Jimena Mora [1], Doina Kulick [3], Nongjian Tao [4], Hugo Destaillats [5] and Erica Forzani [1,2,*]

1 School of Engineering for Matter, Transport, and Energy, Arizona State University, Tempe, AZ 85281, USA; catherinedeng1015@gmail.com (Y.D.); mark.sprowls@asu.edu (M.S.); smora2@asu.edu (S.J.M.)
2 Center for Bioelectronics and Biosensors, Biodesign Institute, Arizona State University, Tempe, AZ 85281, USA
3 Mayo Clinic, Scottsdale, AZ 85054, USA; Kulick.Doina@mayo.edu
4 School of Electrical, Energy and Computer Engineering, Arizona State University, Tempe, AZ 85281, USA
5 Indoor Environment Group, Lawrence Berkeley National Laboratory, Berkeley, CA 94720, USA; hdestaillats@lbl.gov
* Correspondence: eforzani@asu.edu
† Indicates equal contribution and co-first authorship

In order to assure that λ is approximately zero in a closed ventilation setup (RC OFF and Fan set to 0). and that there is no CO_2 generation being introduced in the car cabin from the car's combustion engine, a test was performed as a control experiment with the car engine on, and the ventilation system off completely (RC OFF and Fan set to 0). Figure S1 shows the parameters of temperature, humidity and carbon dioxide levels. Temperature and humidity were similar to the previous car tests, and the carbon dioxide levels did not change significantly from the baseline level recorded right before the car test.

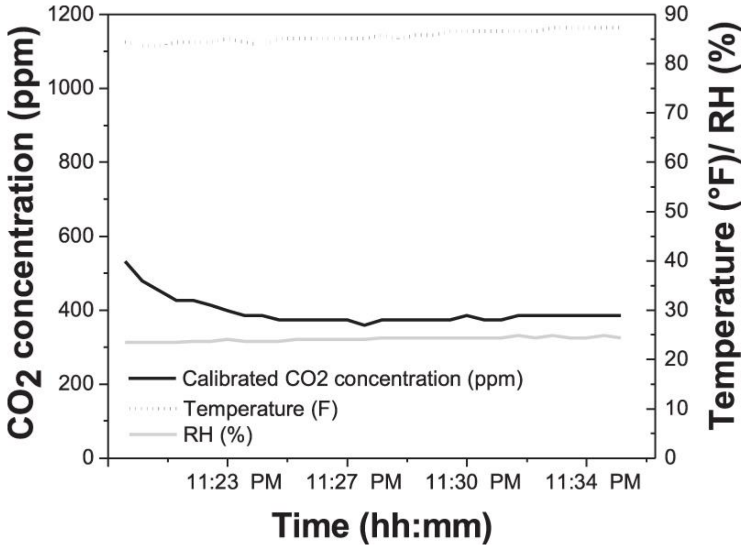

Figure 1. Temperature, relative humidity (RH), and CO_2 concentrations versus time within a car while driving with the ventilation system closed, and the driver breathing in and out of the car cabin via a tubing system that preclude from exhale carbon dioxide build up.

This supporting information includes the detail speed log during the experiment and results for condition #3 (conditions #1-2 described in main text). In this condition, the circulation fan was off during the CO_2 accumulation periods and was turned on for 5 mins after the level reached around 1000-1100 ppm. It is clearly observed that there is a dampening effect with increment of driving speed. At higher speed, the CO_2 accumulated much slower. This may due to the higher-pressure difference between inside and outside when the circulation fan is off, comparing to condition #1. And also, the data of CO_2 concentration decay was very noisy at the highest speed when the circulation fan was on.

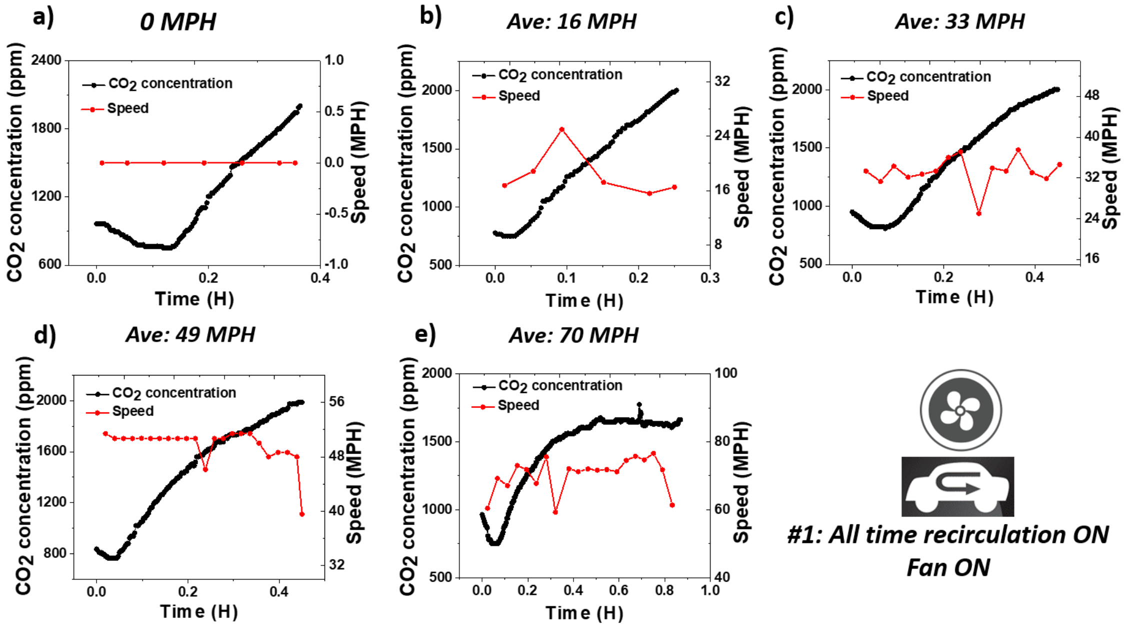

Figure 2. Raw CO_2 monitor results together with real-time speed log for condition #1.

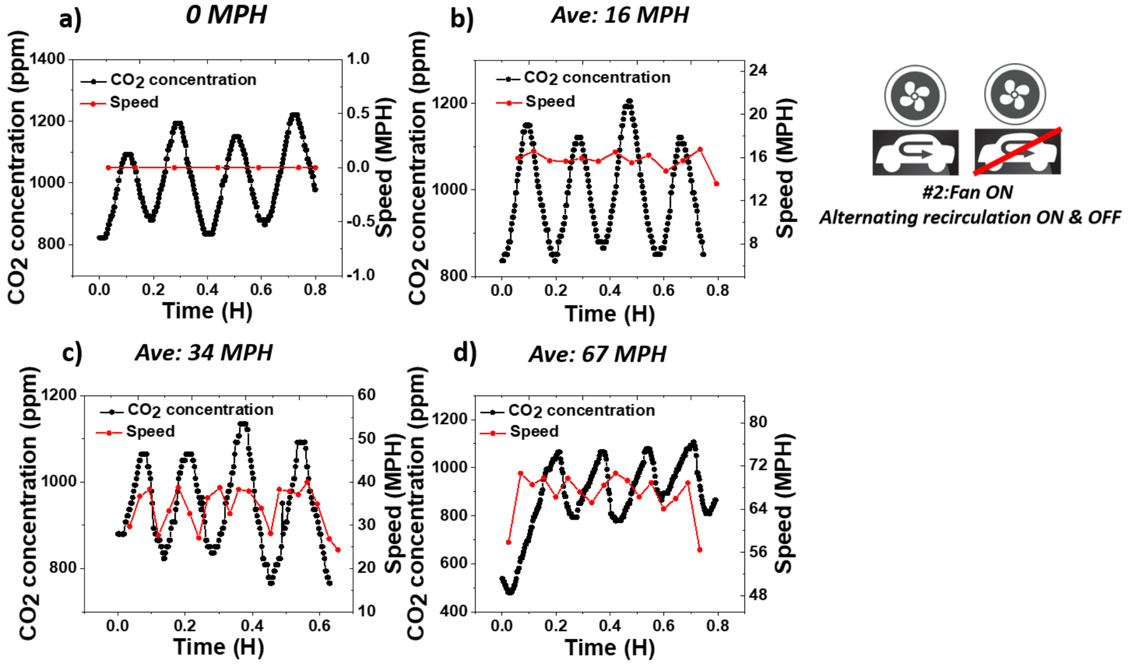

Figure 3. Raw CO_2 monitor results together with real-time speed log for condition #2.

As shown in Fig. S2 and S3, the speed was kept relatively consistent during the tests. This guarantees the low variation of ACH, which relies on speed heavily, as described in main text.
